# Supplementary material for: Are carbon emissions trading and green financial instruments synergistic? -Comprehensive quantitative research based on content analysis
Source: PLoS One. 2024 Mar 7;19(3):e0298601. doi: 10.1371/journal.pone.0298601 (PMC10919601; doi:10.1371/journal.pone.0298601)
Supplement: S1 Data — (ZIP) [file pone.0298601.s001.zip › Data/地级市Z_F工作报告.zip!/╔╜╢1⁄2/╝├─■╩╨/└┤╫╘╣1⁄2╓┌║┼í╛┬φ┐╦╩2╛▌═°í┐_2019.docx]

各位代表：

现在，我代表济宁市人民政府向大会报告工作，请予审议，并请市政协委员提出意见。

一、2018年工作回顾

过去的一年，在省委、省政府和市委坚强领导下，全市上下坚持以习近平新时代中国特色社会主义思想和党的十九大精神为指导，坚持稳中求进工作总基调，贯彻新发展理念，落实高质量发展要求，实施新旧动能转换重大工程，聚焦重点，精准施策，经济社会发展取得新成绩。

（一）发展质量效益持续提升

经济运行稳中向好。预计实现地区生产总值4950亿元、增长6%。一般公共预算收入突破400亿元、增长3.7%，税收占比72.4%、提高3.2个百分点。固定资产投资增长8.5%、居全省第3位。规上工业增加值增长6.5%，高于全省1.2个百分点，营收、利润分别增长8.4%、19%，利润率居全省首位。社会消费品零售总额增长8.6%，拉动经济增长2.9个百分点。城乡居民收入分别增长7%、7.9%。

产业结构持续优化。三次产业调整为9.8：46.2：44.0。战略性新兴产业增长17%、占比提高1.5个百分点。高新技术产业产值增长12%、提高1.2个百分点。高技术制造业增长22%、高于工业15.5个百分点。装备制造业增长8.2%，高于工业1.7个百分点。现代服务业增加值增长15.2%，高于服务业增速7个百分点。粮食生产实现“十五连丰”，新增规上农业龙头企业236家，“金乡大蒜”入选首届中国农民丰收节“100个农产品品牌”，“马坡金谷”贡米获中国国际农产品交易会金奖，27个品牌入选省知名农产品品牌名录。金乡现代农业产业园通过国家验收。

市场主体活力增强。新登记市场主体10.5万户，新增“四上”企业1548家。完成改制企业285家，提前一年完成省定任务。新增中国驰名商标5件。兖矿集团首次上榜世界500强。18家企业入选省“瞪羚”企业，21家企业入选省“隐形冠军”企业，3家企业获评省制造业单项冠军。如意科技入选国家智能制造示范企业，凯赛生物获评工信部制造业单项冠军。2名企业家荣登改革开放40年全国百名杰出民营企业家榜单。

“四新”经济快速成长。新增“四新”经济企业3469家、增长31.8%。工业机器人、集成电路等高新技术产品产量翻番，针状焦、石墨烯等关键核心技术填补国内空白。开展“企业上云进千企”行动，4家企业获评国家“两化”融合贯标试点，6个项目入选省智能制造试点。阿里巴巴、京东、苏宁云商先后进驻，电商交易额2100亿元、增长30%。

（二）新旧动能转换全面起势

项目建设成效明显。建立新旧动能转换重点项目库，市级入库项目605个，开工率99%，完成投资1067亿元；57个项目纳入省项目库，平均单体投资规模16.5亿元。设立新旧动能转换基金14支，总规模177亿元。太阳高档卡纸、华勤卡车轮胎、伊顿汽车零部件等159个项目建成投产。

创新能力不断增强。济宁产业技术研究院挂牌运营，山东大学产研院、黄淮海现代农业研究中心落地建设，如意集团与中科院共建新材料研发中心，倍耐力橡胶轮胎研发中心落户华勤，新增高新技术企业83家，省级以上研发平台47家，入选省级以上人才工程58人，发明专利申请量实现翻番。辰欣药业入选国家技术创新示范企业。邹城市获评科技部首批创新型县市。

对外开放持续扩大。完成进出口总额426.6亿元、增长3.5%。新增出口企业280家，机电产品出口增长22.7%、占比提高9个百分点。新设外商投资企业52家，实际利用外资50.2亿元、增长25.2%。对“一带一路”沿线国家和地区投资20.6亿元、增长40%。首届进口博览会成交合同17.3亿美元，居全省前列。开通兖欧班列，带动出口1.1亿元。

园区支撑能力提升。加快园区体制机制创新，高新区率先实行扁平化管理、全员聘任、岗位工资、绩效考核，6家省级园区探索实行档案封存、薪酬制度改革。“一区一业一所”建设成效明显，4家园区产业研究所建成运行，7家化工园区通过省政府认定、居全省第2位。完成基础设施投资330亿元、增长88.6%。园区增加值、税收分别增长9.2%、13.8%，进出口总额、利用外资占比达到85%、83%。

（三）重点领域改革扎实推进

供给侧改革不断深化。圆满完成年度去产能任务，压减煤炭产能150万吨，减税降费94.7亿元，降低用电成本5.7亿元，规上工业资产负债率下降3.3个百分点，为企业降成本110亿元。

“放管服”改革成效明显。在全省率先实行“市县同权”改革，同权比例达到96%。削减市级行政权力221项。企业注册登记实现“45证合一”。成立行政审批服务局，建成运行市为民服务中心。基本完成政务信息系统整合，市级行政许可事项实现网上办理。

政府机构改革全面实施。坚持转变政府职能，改革机构设置，优化职能配置，加强和完善经济调节、市场监管、社会管理、公共服务、生态保护等职能，成立生态环境、自然资源、市场监管、医疗保障、退役军人事务管理等部门，政府效能持续提升。

体制机制改革实现突破。国有企业改革纵深推进，公司制改制基本完成，市属国有企业全部纳入统一监管。财政体制改革持续深化，绩效目标管理扎实开展，盘活市级存量资金1.5亿元。农村集体产权制度改革全面实施，90%以上的行政村成立新型集体经济组织。监察体制改革基本完成，统计管理体制、镇街管理体制等各项改革稳步实施。

（四）城乡建设呈现新面貌

重点项目加快推进。实施城建重点项目149个、竣工97个。市文化中心高标准建成，图书馆、群艺馆即将开馆，博物馆、美术馆进入展陈施工。打通城区断头路23条。凤凰台公园、儿童公园、市民公园加快建设。圣源大道提升工程、泗河综合开发示范段堤顶路建成通车。济宁大道西延、环湖大道东线顺利实施。火炬路跨线桥、王母阁路跨线桥加力推进。 日兰高速曲阜东互通立交、济徐高速太白湖互通立交开工建设。京杭运河“三改二”、湖西航道升级改造进展顺利。

城市功能日趋完善。改造老旧小区49个，开工棚改6.3万套、建成5.4万套，超额完成省定任务。建成海绵城市15平方公里，实施雨污分流122公里。整修道路136条，整治背街小巷270条。新建公园绿地8个，人均公园绿地面积17.5平方米。新增造林8.8万亩，绿化面积393万平方米。济宁市及曲阜市、邹城市成功创建国家森林城市。

城市品质不断提升。启动新一轮全国文明城市创建，实施 “九大提升”行动，开展“十二项专项整治”，城市文明程度不断提升。实行精细化管理，推行标准化保洁，突出整治“十乱”现象，市县数字化城管平台全部建成。拆除违法建设43万平方米。城市垃圾无害化处理率达到100%，污水集中处理率达到97%以上。

美丽乡村宜居宜业。县域美丽乡村规划实现全覆盖，村庄规划覆盖率达到81%，打造省级美丽乡村示范村42个、市级58个。实施农村“七改”工程，完成农村改厕27万户，危房改造1593户，400个村实现通天然气。开展城乡供水一体化三年行动，建设供水工程26处。新建农田林网32.7万亩。改建农村公路1042公里，镇村公交覆盖率分别达到100%和96.2%。

（五）“三大攻坚战”成果丰硕

风险防控扎实有力。健全风险防控长效机制，“一企一策”破解企业担保圈风险，严厉打击恶意逃废金融债务行为，不良贷款率持续下降。制定政府债务风险化解方案，积极化解存量、严控增量，债务率保持合理水平。

生态环境持续改善。全力抓好中央和省环保督察反馈问题整改，南四湖自然保护区整改工作有序推进。制定实施“四减四增”三年行动方案，关停燃煤机组15万千瓦，“散乱污”“小锅炉”实现动态清零。入选国家北方地区冬季清洁取暖试点市，获中央奖补资金15亿元，完成清洁取暖18.4万户。大气环境质量持续改善，PM2.5浓度均值降至50微克/立方米、居“2+26”通道城市第2位。强化重点流域水污染防治，全面落实河长制、湖长制，国控省控断面全部达标。生态修复成效明显，“大棚房”清理取得阶段性成果，治理塌陷地3.9万亩，修复湿地10.4万亩，治理水土流失9万亩。

脱贫攻坚任务基本完成。各级财政投入资金4.2亿元，发放小额扶贫信贷资金1.05亿元。新建产业扶贫项目113个，扶持贫困户1.7万户、3.4万人，扶贫特惠保险实现贫困人口全覆盖。梁山黄河滩区脱贫迁建工程全面铺开。省定扶贫标准以下贫困人口全部实现脱贫。

（六）民生保障能力不断增强

群众获得感更加充实。民生支出增长8.9%，占全部支出的79%，为民所办十件实事全部完成。人民生活水平不断改善，连续14年提高企业退休人员养老金标准，城乡低保精准救助机制全面建立，救助标准实现“十三连增”。健康济宁扎实推进，市第一人民医院西院区（市中医院新院）建成运营，市立医院一期完成基础施工，建成医联体48个，80%妇幼健康服务机构、90%乡镇卫生院和社区卫生服务中心达到省规范化标准，获评全省医养结合示范先行市。文化惠民成果丰硕，公益演出3.2万场，发放旅游惠民卡5.1万张，新建乡村儒学讲堂1091处，建成村级文化服务中心5955个。第二轮修志全面完成。文化产业增加值增长10%，被评为中国文化竞争力十佳城市。

群众幸福感明显提升。就业创业量质齐升，支出就业创业补贴资金3.5亿元，发放创业担保贷款9.3亿元，新增城镇就业7.7万人，城镇登记失业率2.95%。职工长期护理保险试点圆满完成，居民医保最高报销额度提高到75万元。养老服务日益完善，居家和社区养老服务改革试点通过国家中期验收，新建示范性社区养老服务中心30处，新增养老床位5200张，连续两届进入中国城市公益慈善百强。残疾人康复中心投入使用。优质教育公平普惠，新改扩建中小学校115所，新增学位8.9万个。实施第三期学前教育行动计划，新改扩建幼儿园70所，新增学位7370个，普惠性幼儿园占比达到81.3%。圆满完成“全面改薄”五年规划任务，累计改扩建校舍239万平方米。

群众安全感更有保障。坚守安全发展红线，持续开展“大快严”集中整治，事故起数、死亡人数同比下降40.9%、39.2%。圆满完成上合组织青岛峰会等重大安保维稳任务。社会诚信体系建设扎实推进，获评首批山东省社会信用体系建设示范市。“雪亮工程”全面实施，扫黑除恶专项斗争成效明显，刑事、治安警情同比下降40%、29.7%。全域推进“双安双创”工作，省级食品安全先进市中期评估获省第2名。援疆和扶贫协作取得新成绩。国防动员、人民防空、民兵预备役、国家安全、外事侨务、统计、物价、粮食、地震、体育等工作进一步加强，广播电视、民族宗教、档案、公积金、气象、老龄、残疾人、红十字和妇女儿童等事业取得新进步。

（七）政府自身建设全面加强

持续提高政治站位，树牢“四个意识”，坚定“四个自信”，做到“两个维护”，始终同以习近平同志为核心的党中央保持高度一致。全面落实习近平总书记视察山东、视察济宁重要讲话、重要指示批示精神，坚定不移贯彻党中央、国务院和省委、省政府及市委决策部署。严格落实政府系统全面从严治党主体责任，认真贯彻中央关于意识形态工作决策部署，坚定扛起党的建设和意识形态工作领导责任。持续强化审计监督，全面推进依法行政，自觉接受人大法律监督、政协民主监督和社会各界监督，办理建议157件、提案588件，办复率100%。认真贯彻中央八项规定精神和省市委实施办法，全面抓好中央和省委巡视反馈意见整改，深入开展“大学习、大调研、大改进”，持之以恒转变作风，更大力度推进反腐倡廉，树立起清正廉洁的政府形象。

一年来，全市上下聚焦重点、打通堵点、攻克难点，办成了一些大事、要事、难事，发展定位更加清晰，发展方向更加明确，发展势能更加充沛，为推进高质量发展奠定了坚实基础。我们持续深化战略布局，出台新旧动能转换《实施规划》《实施意见》，确立“1+5+N”总体布局和“5+5”产业体系，编制十大产业专项规划，实施“510”企业培育工程，打造新旧动能转换示范区；大框架开启新一轮城市建设，中心城区确定了“一核引领、两环支撑、三带协同、四城驱动”的“一二三四”重点任务，淮海经济区中心城市建设步伐加快；编制完成乡村振兴战略规划，配套制定5个工作方案，打造乡村振兴齐鲁样板引领区。我们持续强化“双招双引”，组织重大基础设施、华为高峰论坛、北京投资合作、儒商圣城行、宁波生物医药、杭州乡村振兴等系列推介会，精心筹备和参加欧洲、日韩、香港招商活动，落地项目573个，新增院士工作站16个、院士17名，高技能人才2.1万人。我们持续推进重大工程，国务院以国函2号文正式批复我市新机场预可研报告，鲁南高铁全线开工，跨湖高速、董梁高速加快建设，总长106公里的大二环当年立项、当年建设、当年竣工通车。我们持续推进文化“双创”，高水平举办承办央视中秋晚会、尼山世界文明论坛、国际孔子文化节等重大活动，曲阜文化示范区建设拉开框架，尼山圣境一期建成开放，孔子博物馆开馆运行，世界儒学文献中心落成使用，干部政德教育和孔子研究院、孟子研究院、孔子学院总部体验基地建设成效明显，中华优秀传统文化创造性转化、创新性发展取得丰硕成果。我们持续发力攻坚克难，深入开展“项目落实年”活动，建立指挥部作战体系，搭建新旧动能转换可视化督导服务平台，挂图作战，专班推进，“蓝黄红”挂牌督办，发展氛围全面重塑，创业活力全面激发，形成了“说了算、定了干、按期完”的新风尚。发展的实践证明，有壮志、有恒心，一定啃得下硬骨头、打得赢攻坚战！

各位代表，成绩来之不易，这是市委科学决策、坚强领导的结果，是市人大、市政协和社会各界有效监督、大力支持的结果，是全市人民团结拼搏、真抓实干的结果。在此，我代表济宁市人民政府，向全市各族人民，向离退休老领导老同志，向驻济部队、武警官兵、公安干警、消防救援队伍指战员和人大代表、政协委员，向关心支持济宁改革发展的港澳台同胞、海外侨胞和国际友人，表示衷心感谢和崇高敬意!

我们清醒地认识到，发展还面临不少困难和问题，政府工作也存在许多短板和不足。外部环境复杂严峻，世界经贸关系正在重构，全球经济贸易增速趋缓，我国经济运行稳中有变、变中有忧，不确定性增多。我市经济面临下行压力，新旧动能转换不够快，质量效益不够高；产业结构偏重，转型升级任务艰巨；经济外向度偏低，投资增长后劲不足；中心城区支撑能力不强，公共服务水平有待提升；风险防控压力较大，生态治理任重道远，脱贫攻坚还需巩固；民生领域还有不少短板，就业、教育、医疗、居住、养老等与人民群众期盼还有差距；政府职能转变和制度创新仍需深化细化，社会治理水平有待提升；落实全面从严治党责任存在薄弱环节，党员干部中不能为、不敢为、不想为现象还不同程度存在。对这些问题，我们一定以对人民高度负责的精神，勇于担当，敢于负责，加快解决，努力在新一轮发展中当标兵、作示范，跑出好成绩，创造新业绩！

二、2019年主要任务

今年是新中国成立70周年，是全面建成小康社会关键之年。做好今年政府工作，我们要以习近平新时代中国特色社会主义思想为指导，深入贯彻党的十九大和十九届二中、三中全会精神，统筹推进“五位一体”总体布局，协调推进“四个全面”战略布局，坚持稳中求进工作总基调，坚持新发展理念，坚持推动高质量发展，坚持以供给侧结构性改革为主线，坚持深化市场化改革、扩大高水平开放，围绕打造淮海经济区中心城市，深入推进“项目落实年”活动，着力构建“1+233”工作体系，即以强党建为统领，突出抓好动能转换、乡村振兴“两大工作线”，全力突破中心城区、工业园区、曲阜文化示范区“三区”建设，坚决打好风险防控、污染防治、精准脱贫“三大攻坚战”，统筹推进稳增长、促改革、调结构、惠民生、防风险工作，进一步稳就业、稳金融、稳外贸、稳外资、稳投资、稳预期，加快塑造高质量发展新优势，保持经济持续健康发展和社会大局稳定，以优异成绩庆祝中华人民共和国成立70周年。

主要预期目标：地区生产总值增长6.5%左右，一般公共预算收入增长5%左右，固定资产投资增长7%，工业增加值增长7%，城镇登记失业率控制在4%以内，城镇和农村居民人均可支配收入分别增长7%和7.5%，全面完成节能减排降碳约束性指标。重点做好七个方面的工作：

（一）以新旧动能转换重大工程为主线，推动经济高质量发展

主攻十强产业。全面推动“5+5”十强产业规划落地，一个产业、一个智库、一套专班，实施建链、补链、延链、强链工程，重点抓好321个重大产业链项目，提高产业园区化、高端化、集群化发展水平。新一代信息技术产业。以华为山东大数据中心、海富电子、晶导整流器芯片等35个重大项目为牵动，大力发展集成电路、大数据、北斗应用、人工智能等产业，打造信息产业发展高地。高端装备制造产业。着力突破核心技术，加快永生重工、永华数控、海纬机车、珞石机器人等97个重点项目建设，推进5G通讯、工业互联网、物联网等现代服务业与制造业深度融合，支持100家重点企业建设智能工厂和数字化车间，推动济宁制造向济宁智造迈进。新能源新材料产业。重点突破新能源汽车、动力电池、石墨烯、稀土新材料等产业链，依托比亚迪、联诚、圣阳电源等企业引进电机电控等配套项目，支持梁山专用车、重汽商用车加大新能源汽车研发生产，形成整车生产能力；加快泰山玻纤、益大新材料、科伦比恩炭黑等33个牵动性项目建设，推动碳纤维、石墨烯等新材料产业化。节能环保产业。重点发展装配式建筑、固废资源利用等30个产业链项目，壮大融都科技、元泰环保、水发环境、欣格瑞等企业规模，培育发展“绿动力”。医养健康产业。支持如意国际健康城、鲁抗产业园等26个项目加快建设，壮大辰欣、广育堂等骨干企业，构建“医养健智”四位一体医养健康产业链。高端化工产业。依托7家省级化工园区，延伸煤化工、盐化工、橡胶化工、生物化工产业链，强化上下游企业协同，大力推进凯赛生物长链二元酸、中港精细化工、荣信煤化、盛发焦化等54个牵动性项目，建设高端化工产业集聚区。纺织服装产业。强化如意集团龙头带动作用，支持汶上建设休闲服装制造名城，鼓励骨干企业全球配置资源，努力把济宁打造成全球时尚纺织智造基地。现代高效农业。以培育和招引农业龙头企业为抓手，推广“龙头企业+合作社+家庭农场”模式，推进农业与加工业、物流、电子商务、乡村旅游等一二三产融合发展，每个县市区重点打造2-3个优势产业集群。文化旅游产业。支持济宁文旅集团快速发展，提升老景区，培育新景点，拉长吃住行、游购娱、商养学产业链，大力发展全域旅游，努力把文化旅游业打造成新旧动能转换先导产业。现代金融产业。加大金融机构招引力度，培育发展新业态，支持企业挂牌上市，加快科技金融创新区建设，形成现代普惠金融供给体系，实现金融业与实体经济双赢共进。

突破双招双引。树立济宁以外都是外、内资外资一齐抓理念，实施双招双引“一号工程”，以高质量“双招双引”推动高质量发展。明确招商方向。围绕十强产业链，高水平策划包装项目，建立目标客户库、项目库，提高招商精准性、实效性。突出重点区域。深耕以北京为中心的京津地区，以上海、杭州为中心的长三角地区，以深圳为中心的珠三角地区，抓好香港、欧洲、日韩等国家和地区招商，高标准策划系列活动，洽谈推进一批重大项目。创新招商方式。积极对接商会、协会等中介组织，瞄准跨国公司、央企、行业龙头企业、隐形冠军企业，实行园区招商、企业招商、资本招商，形成从项目策划、活动推介，到签约落地、跟踪督导的全链条闭环式招商机制。强化服务保障。坚持要素资源跟着项目走，跟踪抓好签约落地项目调度，重大项目实行台账管理、专班推进、专项督查，保障项目快落地、快建设。抓好引才用才。聚焦产业需求，依托园区、创新平台、科研院所、龙头企业，实施领军人才引进、双创人才集聚等引才计划。完善“一事一议”人才机制，实行“人才一卡通”和服务专员制度，从平台扶持、人才补贴、科研资助等方面制定支持政策，力争新引进高端人才团队10个，入选省级以上重点人才工程15人以上。

强化创新驱动。实施平台倍增计划。做实济宁产业技术研究院，深化与中科院、中国农科院、山东大学产研院等联盟院所合作，加快信息共享平台、研发测试平台、大型设备共享平台建设，支持“校所企”共建中试基地、专业孵化器、大学科技园等协同创新平台，力争新增省级以上创新平台100家。推进产学研融合。强化技术供给保障，打造集政府、企业、高校、科研院所、金融机构于一体的创新共同体，形成全要素创新服务体系。突出企业主体地位。鼓励规上企业建立研发机构，引导企业加大研发投入，支持企业在先进地区、高校集中地和海外建立研发基地、科研中心，力争新认定国家高新技术企业50家以上。

提升园区支撑。强化主导产业培育。每个园区明确2-3个主导产业，围绕主导产业招项目引人才。支持骨干企业建设专业化园区。深化与兖矿、山能战略合作，建设煤炭企业转型升级示范区。强化体制机制创新。突出园区经济管理、投资服务等职能，赋予省级园区县级经济管理权限。推广高新区改革试点经验，实行扁平化管理，全面完成档案封存、全员聘任、薪酬制度等体制机制改革。支持园区实行“管委会+公司”管理模式，引进专业团队运营园区发展，鼓励高新区、兖州工业园建设国际合作园。强化园区招商主阵地。组建专业化招商队伍，专职招商人员不低于园区编制总人数的三分之一。鼓励有条件的园区组建招商公司，引进社会化招商机构，建立第三方招商机制，通过购买服务拓宽招商渠道。强化公共服务配套。加快实施园区基础设施提升三年行动计划，推动基础设施向信息、金融、科技、物流等新“九通一平”跨越。高标准建设教育、医养、专家公寓、职工宿舍等配套设施，提升园区公共服务水平。强化土地集约节约利用。树立“亩产论英雄”导向，实行“标准地”制度，鼓励企业开展“零土地”技改，挖掘低效闲置用地潜力，推动优质资源向优质项目集聚。强化园区争先进位。支持济宁高新区三次创业，推动兖州工业园、邹城开发区创建国家级开发区，力争2家省级开发区进入全省10强，2家进入50强，两年内全部进入前100名。

壮大民营经济。抓好政策落地。全面落实中央和省市支持民营经济高质量发展政策，重点解决配套难、程序多、申报流程复杂等问题，确保惠企政策落地实施。打造强企方阵。实施“510”企业培育工程，支持远山科技、纬世特新材料、神力索具等13家高成长性企业加快发展，启动实施100个重大技改项目，新增“瞪羚”企业10家，隐形冠军企业20家。围绕龙头和高成长性企业，培育一批产业链上下游中小微配套企业，新增“四上”企业1000家以上。实施“十百千”品牌培育工程，新申报中国驰名商标3个。降低经营成本。着力破解融资难、融资贵问题，推行“银税互动”“无还本续贷”制度，降低融资门槛，扩大抵押物范围，设立应急转贷基金，增强企业信贷可获得性。强化企业家培育。提高企业家政治地位，弘扬企业家精神、工匠精神、劳模精神，组织优秀企业家在境内外开展培训，营造尊重企业家、关爱企业家、支持企业家浓厚氛围，打造“亲”“清”新型政商关系。

强化消费拉动。适应消费变化新趋势，从供需两端发力，着力破解有效供给不足难题。积极培育消费热点，增加医疗、养老、家政、文旅、体育等服务供给，鼓励发展定制、体验、智能、时尚等消费新模式，构建消费细分市场。提升消费便利化水平，引进阿里“天猫小店”、京东便利店，开展“绿色消费进社区”“出口产品进商超”等主题活动，推进济宁优质产品中华行、网上行。严厉打击侵权假冒行为，营造安全放心消费环境。

（二）以“一二三四”重点任务为牵动，加快淮海经济区中心城市建设

提升“一核”集聚度。实施总投资376亿元的38个牵动性项目。启动CBD大型商业设施建设，提速推进恒大时代广场、城投综合体、运河金街等重大项目。提升文化、教育、医疗等公共服务水平，全面开放市文化中心，开工建设尚德学校、火炬路中学，建成太白湖幼儿园、市民公园、儿童公园，完成市立医院一期主体工程。启动金融中心规划建设，引进银行、保险、证券等金融机构，打造高端金融商务集聚区。依托济宁医学院、山东理工职业学院等高校资源，加大创新人才和团队引进力度，建设大学科技园、双创基地等科创平台。

打通“两环”关键点。生态环，围绕打造“一环八水绕济宁、十二明珠映古城”大生态格局，完善规划设计，深化城市“双修”，启动平原水库、水利公园、田园综合体建设，开工如意湖、十里湖、九曲湖、龙湖等湿地公园，少康湖建成开园，积极推进国际湿地城市创建。加快塌陷地治理，探索实行“基本农田整理、沉陷区复垦、生态环境修复、湿地景观开发”等模式，建设塌陷地治理示范区。交通环，提速推进24个重大项目，建成鲁南高铁曲临段，完成曲菏段征地拆迁，做好京九高铁梁山站前期工作。开工建设新机场、机场高速，启动南绕城高速规划，连通日兰高速、济徐高速，打造绕城高速路网。加快跨湖高速、董梁高速和济徐高速太白湖互通立交、济宁大道东西延、环湖大道东线、国道327改建等重点工程建设，竣工日兰高速曲阜东互通立交。建成火炬路跨线桥、车站南路跨老运河桥，完成王母阁路跨线桥主体工程并合拢。开工建设城市快速路，修复车站西路，打通洸河路西延、科苑路北延等10条断头路。

强化“三带”协同力。西部以大运河文化经济带为牵动，发挥大运河生态、文化、航运等资源禀赋，推进重大工程项目建设，开建大运河总督署博物馆、济安台古文化街区，建成凤凰台植物公园。提升大运河航运功能，推进主航道“三改二”、湖西航道升级改造、韩庄复线船闸等重点工程。加快兖州国际陆港、任城济杭物流园、梁山物流园、江北现代粮食物流园等临港园区建设。中部以洸府河生态休闲带为轴线，强化生态红线管控，按照景观标准提升河堤绿化水平，增加便民服务设施，打造城市生态休闲景观带。东部以泗河绿色发展带为廊道，完成拦河闸坝、泗河堤顶路建设，实施滨河生态绿化，发展特色观光农业，推动沿线工业板块绿色发展，形成东部工业经济集聚区和乡村振兴示范带。

打造“四城”新引擎。蓼河新城，以服务创新创业为定位，实施鸿雁湖、景云湖等生态工程，高标准建设麒麟岛、红星爱琴海等4个城市综合体。运河新城，以发展智慧产业为定位，启动梁济运河西岸绿化、老运河修复，抓好中兴产业园、中科智造等项目建设，在优化生态环境、提升产业层次上实现新突破。西部新城，以先进制造业集聚区为定位，聚焦基础设施和产业项目两大重点，加快推进新能源汽车产业园、中关村京港澳双创中心等重大项目。颜店新城，完成总规、控规和专项规划，实施子渊湖、双创展示中心、宁安大道、鲁王路等13个重点项目建设，全面拉开新城框架。

推进智慧城市建设。以创建全国文明城市为抓手，提升城市建设管理智慧化水平，打造精致济宁、魅力济宁。完善智慧城市顶层设计。整合数据资源，建设全市统一的人口、法人、地理空间等基础数据库，完善数字管理信息系统、地下管线综合系统，加快5G网络、光纤网络等基础设施布局，形成“建设一张图”。实施智慧交通工程。推动大数据、云计算在交通领域应用，新建智慧公交站台、电子站牌60个，提升重点路段交通智慧管控水平。实施公共停车场建设三年行动计划，新建立体智能停车场6座，努力解决停车难问题。提升智慧城市管理水平。建设济宁城管云平台，推进一二级平台联网对接，加快智慧市容、智慧环卫、智慧市政、智慧园林等平台建设，构建智慧化城市管理体系。加快智慧民生建设。推动智慧旅游、智慧教育、智慧医疗、智慧警务、智慧民政建设，完善济宁旅游APP，提升远程医疗、双向转诊等医疗服务智能化水平，让群众享受到智慧城市带来的便利。

（三）以乡村振兴“五大行动”为抓手，打造齐鲁样板引领区

实施产业振兴行动。开展粮食绿色高质高效创建，确保粮食总产稳定在100亿斤以上。大力发展特色有机农业，推动南四湖渔业绿色生态养殖。壮大农业龙头企业，优选50家企业重点扶持，提升嘉冠油脂、百盛生物、绿源食品、菱花味精等本土企业市场竞争力，支持今麦郎、娃哈哈、盼盼食品、益海嘉里、禾丰牧业、无穷食品等企业做大做强。积极培育新型农业经营主体，完善农民返乡创业政策，加强职业农民、农村致富带头人培训，推进泗水、鱼台国家电子商务进农村综合试点，新认定示范合作社、家庭农场100家，土地流转率达到40%以上。强化农产品品牌培育，加大“济宁礼飨”区域品牌推广，新认定“三品一标”80个，创建国家级农产品质量安全市。重点打造金乡大蒜、微山渔湖产品、任城苗木、鱼台大米、高新区花卉等14个农产品专业市场。支持泗水建设全国农村一二三产融合发展先导区。发展壮大村级集体经济，年内消除1000个以上集体收入3万元以下的薄弱村。

实施环境提升行动。启动乡镇驻地“十个一”工程，推进城乡供水一体化，完成7处水厂建设，规模化供水覆盖率达到75%以上，提升70.5万人饮水安全。编制农村污水治理专项规划，配套完善镇村生活污水处理设施，开展农村生活垃圾分类试点。实施农村“七改”工程，水电暖气网并进，改造“四好”农村路800公里，在基本完成天然气“镇镇通”基础上，推动供气设施向农村延伸，完成330个村建设任务。推进美丽乡村标准化建设，加强镇驻地环境卫生综合整治，提升农村生产生活环境。

实施绿满乡村行动。加强农田林网、河湖岸线、道路两侧、农村庭院等重点区域绿化，推进荒山、空地绿化全覆盖，建设森林乡村示范村100个、重点村700个，新造林15万亩。加快泗水圣水峪、微山岛栖凤园、邹城云梦桃源小镇等田园综合体建设，扶持发展乡村旅游，培育5个特色旅游小镇、30个景区村庄。

实施乡村善治行动。强化村级党组织政治引领、组织发动、服务群众和社会治理能力。深化乡村法治建设，借鉴“枫桥经验”，创新群众工作方法，促进农村和谐稳定。推进“乡村记忆”工程，建设非物质文化遗产传承体验基地10个。

实施示范引领行动。研究制定齐鲁样板引领区标准体系，分类规划、分类定策、分类推进。重点打造200个示范村、20个示范镇，加快运河文化带、泗河生态带、尼山片区、微山湖片区、黄河滩区“两带三区”建设，打造一批集“五大振兴”于一体的示范项目。

（四）以深化改革扩大开放为动力，增强竞争新优势发展新活力

深化经济体制改革。大力推进供给侧结构性改革，完成省定煤炭压减任务。调整完善财政运行体制，统筹“全口径”预算编制和专项资金安排，全面实施预算绩效管理。加快地方金融监管体系建设，强化金融监管职能。深化国有企业治理结构、产权结构、组织机构改革，完成县属国有企业统一监管，建立现代企业制度，推进国有企业混合所有制改革。

深化“放管服”改革。全面落实“3330”工作机制。瞄准难点，深化市县同权改革，治理中介乱收费，降低企业经营成本和准入门槛。聚焦堵点，完善市县乡村四级政务服务体系，逐步实施无差别一窗受理一链办理，打通“数据孤岛”，构建“政务服务一张网”。围绕弱点，加强事中事后监管，推行跨部门联合监管和“互联网+监管”。紧盯重点，优化企业开办、不动产登记、建设项目审批、水电气暖报装等关键环节，推进审批服务标准化、规范化。

深化重点领域改革。全面落实市级机构改革实施意见，优化政府机构设置和职能配置，有序做好转隶、集中办公等工作。深入实施农村集体产权制度改革，开展农村宅基地有偿退出机制和“三权分置”试点，加快集体建设用地盘活利用。完善“三医联动”运行机制，深化医疗价格、薪酬制度、医保支付方式等改革。统筹推进县域城乡义务教育一体化改革，扎实做好行政执法、事业单位等重点领域改革。

打造对外开放新高地。提升利用外资水平。精心筹备香港山东周和欧洲、日韩招商活动，高水平承办第18届泛黄海中日韩经济技术合作交流会；在北京、上海等跨国公司驻华总部集中城市，策划系列登门拜访洽谈活动；抓住我省在青岛举办跨国公司峰会机遇，组织跨国公司高管济宁行。全心全意做好已落户济宁外资企业服务保障，鼓励企业增资扩股。深化与华为、华润、光大国际等企业合作。积极开拓外贸市场。鼓励120家重点外贸企业扩大出口规模，稳定外贸发展基础。持续扩大外贸经营队伍，新增进出口企业200家以上。精心组织“千企百展”“跨境电商进万企”活动，为企业提供多元化市场渠道。加快培育公共服务平台、海外仓，扎实推进济宁综合保税区建设。鼓励企业“走出去、引进来”双向发力。支持有条件的企业到境外并购知名品牌和营销网络，布局生产加工基地、研发设计中心。推动优势企业与国外隐形冠军企业合作，实行境外研发、境内生产，鼓励走出去企业返济投资。培育本土跨国公司。

（五）以曲阜文化示范区建设为引领，打造文化强市首善之区

建设世界文明交流互鉴高地。扎实推动曲阜文化示范区建设规划落地实施，编制示范区核心区空间规划，抓好“四大平台”建设，筹建中国儒商学院，打造世界儒学中心。强化尼山片区核心功能，实施尼山世界文明论坛提升工程，开工建设尼山会堂、鲁源小镇等重点项目，推动形成“南有博鳌、北有尼山”大格局。办好国际孔子文化节、世界儒学大会等重大活动，推进孔子学院总部体验基地、政德教育学院基地、儒学人才高地建设。

推动文旅融合深度发展。整合文化旅游资源，打造拳头产品，策划精品路线，提升文化旅游综合实力。拓展文化旅游市场，加强与国内百强旅行社合作，在扩大省内市场的基础上，北上京津冀、南下江浙沪、西拓中原腹地，在北京、上海、郑州、南京、杭州等中心城市举办文旅推介会；发挥儒家文化优势，积极拓展日韩、东南亚等海外市场，增强文化旅游消费对经济发展的拉动力。挖掘运河文化内涵，规划建设运河文化特色街区，开通济宁城区至微山湖水上旅游专线。支持微山湖、水泊梁山、孟府孟庙争创5A级景区。

健全公共文化服务体系。扎实推进新时代文明实践中心建设，建成县实践中心、乡镇实践分中心。管好用好基层文化设施，提升县级图书馆、文化馆服务水平。推动优秀传统文化“六进”普及，推行“图书馆+书院”、孔子学堂等公共文化服务模式，新建乡村儒学讲堂100处。开展省市县联合购买公共文化服务试点工作。创作推出一批文艺精品。常态化开展送戏进农村、进校园活动。

打造思想道德建设高地。广泛开展精神文明创建活动，提升文明单位、文明校园、文明行业创建水平，打造彬彬有礼文明城市。深化“四德工程”示范创建，评选一批道德模范、济宁好人，让道德文明内化为市民自觉行动。

（六）以打赢“三大攻坚战”为重点，保持全市大局和谐稳定

防范化解重大风险。强化金融风险预警，健全监测防控体系。突出抓好企业担保圈风险防控，大力化解不良贷款，严厉打击恶意逃废金融债务行为。规范政府举债融资，有序处置存量政府债务，妥善解决在建项目后续融资，防止金融、财政、债务三险叠加。严格安全生产责任制，落实“三个必须”要求，强化安全风险管控，加强双重预防体系建设，坚决遏制重特大事故发生。

提高生态环境质量。坚定不移抓好中央和省环保督察反馈问题整改，不折不扣完成年度整改任务。扎实开展“四减四增”专项行动，整合关停岱庄电厂2台机组和辰光电厂2台锅炉，改造气代煤电代煤15万户，新增集中供暖面积300万平方米，力争完成公交车、出租车、市政车辆清洁能源全替代，PM2.5、PM10浓度分别下降2%、5.8%以上。打好碧水保卫战，积极推进河长制湖长制从“有名”向“有实”转变，完成河湖管护范围划定，加强饮用水源地监管，强化农业面源污染防治，确保重点河流水质稳定达标。加快固废处置设施建设，建成运营济宁中科、泗水、邹城、金乡、嘉祥5个生活垃圾焚烧发电厂，开建兖州、微山、鱼台、梁山、汶上5个垃圾焚烧发电厂，全市生活垃圾日处理能力达到5000吨以上。

巩固提升脱贫成果。加大20个重点乡镇、200个重点村扶贫力度，抓好黄河滩区贫困人口脱贫攻坚，完成迁建民生项目主体工程。统筹开发式扶贫与保障性扶贫，建立稳定脱贫长效机制，让贫困家庭真脱贫、不返贫。

（七）以让人民生活得更美好为目标，加强社会事业建设办好民生实事

多措并举稳就业。实施援企稳岗护航行动、高校毕业生就业创业促进计划，多渠道开发稳定就业岗位，新增城镇就业7万人，扶持创业1.5万人。加强高校毕业生、失业人员等重点人员培训，加快建立全方位公共就业服务体系，消除“零就业”家庭。

优先发展教育事业。深入实施学前教育三年行动计划，新改扩建幼儿园40所，新增学位9000个。持续推进解决大班额问题，新改扩建学校30所，新增学位2万个，启动原济宁师专旧址改造。提升教育信息化水平，建成智慧校园20个、智慧课堂100个，网络名师工作室30个。积极发展职业教育、特殊教育，支持驻济高校加快发展。高质量承办山东省第十四届中学生运动会。加强师德师风和专业能力建设，实施万名教师培训计划，选树100名“师德模范”，打造高素质教师队伍。

提升社会保障能力。全面推行职工长期护理保险，提高城乡低保和农村特困人员救助供养标准，积极推进重特大疾病救助和临时救助工作。提升乡镇敬老院服务能力，完成139处乡镇敬老院改厨、改浴、改厕任务。建立城乡居民养老保险正常调整机制，适度提高离退休人员养老保险待遇、居民医保财政补助标准。实施政府灾害救助保险项目，提高孤儿和困境儿童保障能力。建成启用示范性社区养老服务中心30个、社区老年人食堂50个，新增养老床位5000张，打造全国居家和社区养老服务改革样板。

加快健康济宁建设。启动基层医疗卫生服务能力提升三年行动计划，改造薄弱乡镇卫生院13处，招聘乡镇卫生院专业技术人员300人以上。加快市第一人民医院高新区分院二期工程、市第二人民医院济北院区、济宁卫生学校新校区建设。完善分级诊疗制度，扎实推进县域医共体“六统一”管理。提升市中医院办医水平，打造20家精品“国医堂”。广泛开展全民健身活动。扎实推进省医养结合先行市建设。积极创建国家食品安全示范市。

加强社会治理创新。抓好全国城市管理信用体系试点市建设，探索实行政务、企业、个人诚信“红黑名单”制度，打造“信用济宁”品牌。完成第四次经济普查。深入开展扫黑除恶专项斗争，积极化解社会矛盾，深化人民调解“三易三零”创建活动，实现政府治理和社会调节、居民自治良性互动。强化舆情监管，提升舆论引导能力。加强国防动员、人民防空、民兵预备役工作，争创全国双拥模范城“八连冠”。

三、全面加强政府自身建设

做好新时代政府工作，必须加强政府自身建设，时刻不忘初心、牢记使命，做到信念过硬、政治过硬、责任过硬、能力过硬、作风过硬，努力向全市人民交出满意答卷。

增强政治定力。坚持把党的政治建设摆在首位，强化理论武装，提高政治站位，树牢“四个意识”，增强“四个自信”，做到“两个维护”，始终同以习近平同志为核心的党中央保持高度一致。大力推进中央和省委巡视反馈意见整改工作，确保反馈问题整改到位。严格落实意识形态工作责任制，加强政府系统意识形态阵地管理，坚决捍卫意识形态领域安全。严明党的政治纪律、政治规矩，坚定不移把党的领导贯彻到政府工作各个方面，全面落实中央和省市委决策部署。

提升思想境界。破除固步自封、安于现状、畏难发愁的消极思想，摒弃资源依赖的思维定势、因循守旧的求稳心态，以敢为人先的闯劲和魄力，突破发展难点，消除民生痛点，疏通市场堵点。时刻保持危机意识、赶超意识和责任意识，全面提升发展理念、境界标准和工作水平。大力推进制度创新，始终保持争先进位、争创一流的精神状态，以思想新境界提升发展新水平。

加强法治建设。严格遵守宪法法律，依法办事，依法用权，把政府工作全面纳入法治轨道。坚持科学、民主、依法决策，严格执行重大行政决策法定程序，对涉及重大公共利益、群众切身利益的事项，广泛听取各方面意见，真正使政府工作体现人民意愿。全面落实行政机关负责人出庭应诉制度。认真执行市人大及其常委会的决议决定，自觉接受人大法律监督、政协民主监督、社会和舆论监督，做好人大建议、政协提案办理工作。全面推行政务公开，让权力在阳光下运行。坚持诚信为本，言必信、行必果，建设法治政府、诚信政府。

提高政府执行力。坚持“干字当头、实字为先”，不做表面文章，不搞形象工程，不弄虚作假，不说大话空话，以踏石留印、抓铁有痕的韧劲，认真负责地抓好落实，确保定一件干一件，干一件成一件。深化整治形式主义、官僚主义成果，坚决杜绝空泛表态、应景造势、敷衍塞责、出工不出力。优化指挥部运行机制，完善挂图作战体系，推动重大工程、重点项目、重大事项落地实施。规范督导检查，完善考核问责机制，坚决纠正“层层压实责任，层层不负责任”问题。全面推行分级分类培训，增强政府工作人员的群众观点、法治思维、创新意识和专业能力，培养高素质干部队伍。

做到清正廉洁。坚定扛起全面从严治党政治责任，以永远在路上的执着，加强政府系统廉政建设。盯紧重大工程、重点领域、关键岗位，强化权力集中、资金密集、资源富集部门监管。持续整治群众身边腐败和作风问题，开展精准脱贫、教育医疗、环境保护、食品药品安全等侵害群众利益问题专项整治。主动接受纪检监察监督，深化源头治理，形成靠制度管权、管事、管人的长效机制。加强纪律教育，引导党员干部坚定理想信念，强化宗旨意识，永葆为民、务实、清廉的政治本色。

各位代表，使命呼唤担当，奋斗创造幸福。让我们更加紧密地团结在以习近平同志为核心的党中央周围，深入学习贯彻习近平新时代中国特色社会主义思想，在中共济宁市委的坚强领导下，真抓实干，埋头苦干，奋力开创新时代济宁改革发展新局面，以优异成绩庆祝中华人民共和国成立70周年！
